# Preparation and Characterisation of Poly(methyl metacrylate)-Titanium Dioxide Nanocomposites for Denture Bases

**DOI:** 10.3390/polym12112655

**Published:** 2020-11-11

**Authors:** Mariusz Cierech, Marcin Szerszeń, Jacek Wojnarowicz, Witold Łojkowski, Jolanta Kostrzewa-Janicka, Elżbieta Mierzwińska-Nastalska

**Affiliations:** 1 Department of Prosthodontics, Medical University of Warsaw, 02-006 Warsaw, Poland; marcin.szerszen@wum.edu.pl (M.S.); jkostrzewa@wum.edu.pl (J.K.-J.); emierzwinska@wum.edu.pl (E.M.-N.); 2 Institute of High Pressure Physics, Polish Academy of Sciences, 01-142 Warsaw, Poland; jacek.wojnarowicz@tlen.pl (J.W.); w.lojkowski@labnano.pl (W.Ł.)

**Keywords:** denture bases, nanocomposites, poly(methyl methacrylate), titanium dioxide nanoparticles (TiO_2_ NPs), polymerisation time, colour

## Abstract

Introduction of titanium dioxide nanoparticles (TiO_2_ NPs) to poly(methyl methacrylate) (PMMA) aims to improve the mechanical, microbiological and tribological properties of dental prosthesis bases. The aim of the research was to assess the polymerisation time and the change in the colour of the new biomaterial. Samples with the 1 wt% and 2 wt% content of TiO_2_ additionally modified by ultrasounds were created. The effectiveness of ultrasounds was assessed by comparing the average size of conglomerates in a liquid acrylic resin monomer by means of a dynamic light scattering (DLS) analysis. The biomaterial structure was assessed by the energy-dispersive X-ray spectroscopy (EDS) analysis. The colour change was analysed by means of a colorimetric test and provided in the CIE (Commission internationale de l’éclairage) L*a*b* and RGB (Red Green Blue) colour palette. It was observed during the DLS test that the ultrasonic homogenisation process caused an increase in the suspension heterogeneity. The EDS analysis confirmed the presence of nanoparticles sized below 100 nm, which constitutes a ground for calling the new biomaterial a nanocomposite. The addition of TiO_2_ NPs as well as the ultrasounds result in the reduction of the average PMMA polymerisation time. The obtained data reveal that the addition of both 1 wt% and 2 wt% causes a considerable change in the PMMA colour: its whitening. To summarise, the reduced polymerisation time of the new biomaterial fully enables performance of standard procedures related to creation of dental prosthesis bases. Due to the considerable change in the colour, the clinical application is limited to performance of repairs or relining of the prosthesis, where the new material is located in an unaesthetic zone.

## 1. Introduction

A consequence of an ageing society is the growing number of entirely or partly toothless patients. In such situations, despite the progress and the increasing availability of implant treatment, the most frequently performed restorations are full or partial tissue supported dentures with a large base. Such kinds of restorations together with favourable systemic and local factors predispose to inflammations of the mucous membrane of the denture-bearing area—denture stomatitis [1]. The material that is most frequently used for making dentures is poly(methyl methacrylate) (PMMA), which has a range of properties that facilitate the accumulation of the bacterial and fungal biofilm on its surface [2,3]. For this reason, attempts are made continually to modify the chemical composition of PMMA by adding substances characterised by antifungal and antibacterial action. The use of nanoparticles, among others of silver (Ag), titanium dioxide (TiO_2_), zinc oxide (ZnO) or platinum (Pt) with proved properties inhibiting the formation of bacterial and fungal biofilm on the PMMA surface, has been reported numerous times [4,5,6,7,8,9,10,11,12]. However, there are no clinical tests confirming the effectiveness of thus created nanocomposites in preventing or treating denture stomatitis. There are many reports about the modification of thermally polymerised acrylic materials with TiO_2_, while there are relatively few of them about self-curing polymers [4,5,6]. The presence of a chemical catalyst may affect the quality of the distribution of filler in PMMA’s volume. This paper is a continuation of the earlier publication of the authors [13], which characterised titanium dioxide nanoparticles (TiO_2_ NPs) before their potential use for PMMA modification. An optimum situation would be the incorporation of titanium dioxide nanopowder with the use of standard equipment of a dental practice or a dental technician’s laboratory. The methods of mixing titanium oxide in a monomer suspension or mixing TiO_2_ powder with resin powder, described in the literature, are often streamlined by using additional devices that are supposed to increase the filler’s dispersion. Such devices include, among others, laboratory ultrasonic mixers, shakers, specialist mortars or high energy ball mills [6,14,15]. The introduction of a substrate mixing procedure with the use of the aforementioned devices seems to be very difficult, though, in the conditions of a dental practice or a dental technician’s laboratory. Therefore, the authors decided to check the potential use of an ultrasonic cleaner, which is standard equipment of every such place. Another aspect of the utility features of the new biomaterial is its colour, and hence the possibility to make a prosthesis with satisfactory aesthetic qualities. The denture base covers or reconstructs the missing anatomical structures such as hard palate, alveolar ridge, or gums. Very often, these are elements are responsible, to the same extent as the teeth shape and positioning, for the final aesthetic effect of the work. The growing demands of the patients treated with the use of a prosthesis are the reason why restorations must meet the highest aesthetic requirements. Thus, the addition of a nanofiller must not deteriorate the features of gum imitation by the acrylic material. The restoration colour is undoubtedly of greatest importance, because it must be fully acceptable both by the patient and by the doctor.

The aim of this paper is to introduce TiO_2_ nanoparticles to a chemically polymerised acrylic material and to test selected properties (polymerisation time and colour) of thus created nanocomposite depending on the manner of nanoparticle incorporation.

## 2. Materials and Methods

### 2.1. Materials

Titanium dioxide nanoparticles with the catalogue number 637254-50g and the purity of 99.7% produced by Sigma-Aldrich (Saint Louis, MO, USA). TiO_2_ NPs were not subject to any purification or modification processes.

### 2.2. Methods of NP Characterisation

The characterisation of the TiO_2_ samples presented herein was performed at an accredited research laboratory with certificate no. AB 1503 (Laboratory of Nanostructures, IHPP PAS, Warsaw, Poland), which operates in accordance with PN-EN ISO/IEC 17025:2018-02 [16]. The procedures of TiO_2_ NPs tests were described in detail in the earlier papers of the authors [8,13,17].

The phase composition of the NP samples was determined by the X-ray diffraction method (XRD) (X’Pert PRO, copper lamp (CuKα), Panalytical, Almelo, Netherlands). The diffraction analyses were performed with the measurement step of 0.02° within the range of 2 theta angle from 10° to 100°, at room temperature. The average crystallite size was determined with the use of Scherrer’s formula [18] and the Nanopowder XRD Processor Demo web application [19].

The skeleton density (pycnometric density) was determined using the helium pycnometer (24 ± 1 °C, ISO 12154:2014, AccuPyc II 1340, FoamPyc V1.06, Micromeritics^®^, Norcross, GA, USA). The specific surface area was determined by the nitrogen (99.999% N_2_) adsorption method based on the linear form of the BET (Brunauer–Emmett–Teller) isotherm equation (ISO 9277:2010, Gemini 2360, V 2.01, Micromeritics^®^, Norcross, GA, USA). The average size of NPs was calculated based on the obtained density and specific surface area results, assuming [8,13] that the samples are composed only of spherical and identical particles.

The tests of NPs morphology were performed using the scanning electron microscope (SEM) ULTRA PLUS (ZEISS, Germany) and transmission electron microscopy (TEM) (Talos F200X, Thermo Scientific™, Waltham City, Massachusetts, MA, USA).

The average size of TiO_2_ particles in a water suspension was measured with the use of the Zetasizer Nano-ZS ZEN 3600 analyser produced by Malvern Instruments Ltd. (Malvern, UK) in accordance with ISO 22412:2008 standard using dynamic light scattering. An Energy Dispersive X-ray (EDS) (Quantax 400, Bruker, Billerica, MA, USA) detection system built in the SEM instrument was used to record the X-ray emission spectra of the samples interacting with the electron beam.

### 2.3. Preparation of Poly(methyl methacrylate) Modified with TiO_2_ Nanoparticles

A chemically polymerised acrylic material was used for the test (Duracryl Plus; Spofa Dental, Jicin, Czech Republic). The powder–liquid volume ratio recommended by the producer is 3:1, which corresponds to 22 g of the polymer and 10 mL of the liquid monomer. After mixing the components and achieving the acrylic dough phase, the material was placed between two glass plates to obtain a thin material layer for the SEM test. Further polymerisation was carried out in a pressure polymeriser (Zhermack 7L TM, Badia Polesine, Italy) at a temperature of 65 °C and under the pressure of 2 bar for 30 min. Thus created samples were the control group. During the nanocomposite preparation, first of all, the calculated quantity of titanium dioxide nanopowder (Table 1) was mixed manually in a liquid acrylic resin monomer for 60 s and subsequently, the calculated quantity of the polymer was added to obtain a nanocomposite containing 1 and 2 wt% of TiO_2_ NPs. Subsequently tested groups were samples with the use of ultrasounds for potentially better dispersion of nanoparticles in the acrylic resin monomer. After adding the nanoparticles to the monomer and mixing them for 60 s, the vessel was placed in an ultrasonic cleaner (Jeken PS-06A, Dongguan, China) with a power of 35 W and the frequency of 40 kHz for 180 s. The control group was composed of the monomer without nanoparticles, placed in an ultrasonic cleaner. The further procedure was not changed. 

### 2.4. Measurement of Initial Polymerisation Time of Modified PMMA

In order to compare the initial polymerisation time of the modified or unmodified chemically polymerised acrylic material, the material for testing was divided into six equinumerous groups described in Table 2. Four trials were carried out for each group. Polymerisation time was registered using a digital stopwatch from the moment of the beginning of the addition of the polymer powder to the liquid monomer (0% and 0%S group) or to the liquid monomer with the TiO_2_ NPs content (monTiO_2_ NPs) (1%, 1%S, 2%, 2%S groups) until the moment when the compound was no longer viscous (beginning of the acrylic dough phase) [20]. In all trials, the first 60 s were devoted to manual mixing of the powder with the monomer or monTiO_2_ NPs with the use of a stainless steel spatula. While adding the polymer to the monomer (0%S) or monTiO_2_ NPs (1%S and 2%S), the 0%S, 1%S and 2%S groups were additionally placed in an ultrasonic cleaner (Jeken PS-06A, China) with the power of 35 W and the frequency of 40 kHz during the 60 s mixing with the spatula. The subsequent phases of the initial polymerisation took place in a closed glass vessel. 

### 2.5. Comparison of Colour of Modified and Unmodified PMMA 

In order to compare the colour of the obtained material with the colour of the unmodified chemically polymerised acrylic material, the material prepared during the measurement of the initial polymerisation time was used and three cylindrical samples sized 30 mm × 3 mm were prepared with the use of a polystyrene mould, one sample from each type of the obtained material (groups 0, 2, 4). Subsequently, the samples were subject to a colorimetric test with the use of a digital colorimeter (ColorReader, Datacolor AG Europe, Rotkreuz, Switzerland) connected via the wireless Bluetooth interface with the dedicated software. The data obtained from the equipment’s software are recorded in the L*a*b* standard introduced by the International Commission on Illumination (CIE—Commission internationale de l’éclairage). The equipment is fitted with a light source being 6 LEDs, and the parameters being the observation angle—10°, and the illuminant—D65, are consistent with the standards accepted by CIE. The CIE L*a*b* system permits a colour description in a three-dimensional space, where the coordinates are determined by three constituents: “L” axis—brightness (0—black, 100—white), “a” axis determines the amount of red and green colour (positive values—red, negative values—green), while “b” axis determines the amount of yellow and blue colour (positive values—yellow, negative values—blue). Each sample was subject to three independent measurements in various places of the sample and the results were recorded in the CIE L*a*b* and RGB colour space. The RGB colour space model is one of the most often used models for assessment of colours analysed in photographs, computer graphics or digital displays. It consists in an additive synthesis of three constituent colours: R—red, G—green, B—blue, and the appropriate projection of three light beams mixed in defined proportions results in the creation of a wide range of secondary colours. The colour record in the RGB model is composed of 3 successive numbers within the range from 0 to 255, where 0.0.0 means black, and 255.255.255 means white. In order to compare the variance of the obtained colours, the equation proposed by CIE in 1976 was used: $∆E=\sqrt{{\left(∆L\right)}^{2}+{\left(∆a\right)}^{2}+{\left(∆b\right)}^{2}}$, which shows the numerical distance between two colours in the three-dimensional space of the CIE L*a*b* system [21].

### 2.6. Statistical Analysis

The data were evaluated for normal distribution using the Kolmogorov–Smirnov test. Then after checking the homogeneity of variance (Brown–Forsythe and Levene assays) the Student’s *t*-test for independent samples was performed. The level of significance was established at a *p* value = 0.05. All data were computed using the Statistica 10.0 program (StatSoft, Inc. Tulsa, OK, USA).

## 3. Results

### 3.1. Characteristics of Nanoparticles

**Figure 1:**
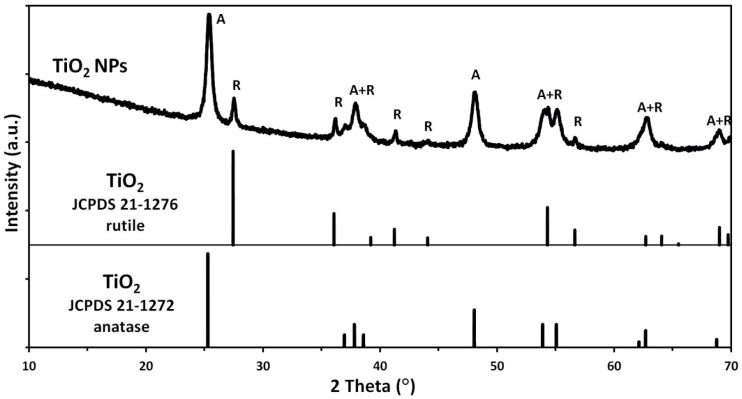
X-ray diffraction pattern of TiO_2_ nanoparticles (NPs).

**Figure 2:**
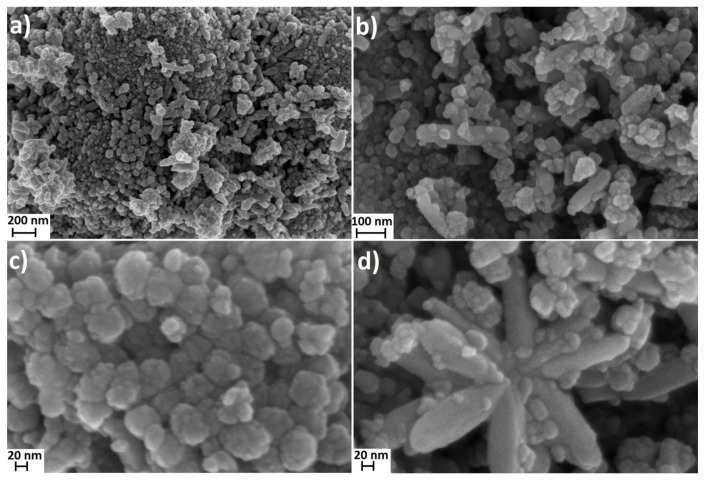
SEM images of NPs: (**a**–**d**) TiO_2_.

**Figure 3:**
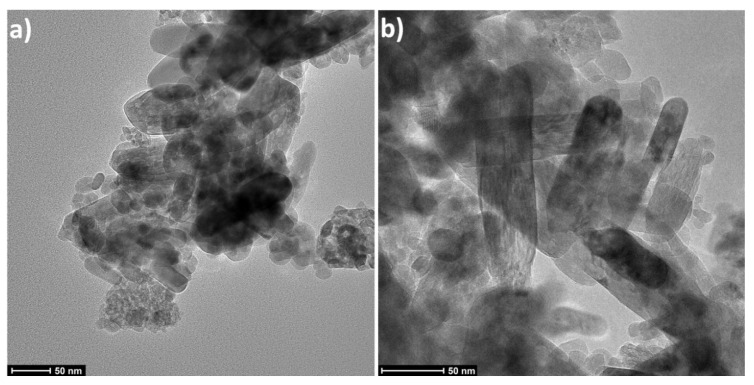
TEM images of NPs: (**a**,**b**) TiO_2_.

The X-ray powder diffraction indicated that the TiO_2_ sample was composed of a mixture of two crystalline phases of TiO_2_: anatase and rutile (Figure 1). In the sample, the presence of TiO_2_ in the form of the anatase phase prevailed. The primary fraction in the sample was spherical/oval TiO_2_ particles sized from 10 to 70 nm (Figure 2). SEM and TEM (Figure 3) images indicated also the presence of TiO_2_ particles in the form of rods with a thickness of approx. 20–40 nm and the length from 50 nm to 200 nm. TiO_2_ nanorods occurred in the form of single objects and formed star-shaped aggregates (Figure 2d). Based on the XRD tests it can be stated that the primary fraction of spherical/oval-shaped TiO_2_ particles is anatase, while rod-shaped TiO_2_ particles are rutile.

The summary of the characteristics of the TiO_2_ NPs sample is presented in Table 3. The theoretical density of TiO_2_ in the form of anatase is 3.79 g/cm^3^, while that in the form of rutile is 4.13 g/cm^3^. The density of the TiO_2_ NPs sample was 3.68 g/cm^3^. The difference between the theoretical value and the actual value of nanomaterial density is well known in the literature and may arise from several causes, e.g., presence of crystal lattice defects (imperfection of crystals), nonstoichiometry of TiO_2_, presence of impurities, e.g., OH groups, presence of closed pores, presence of an amorphous phase [18]. The average nanoparticle size calculated as a result of the conversion of the density and specific surface area results was 20 nm, while the crystallite size calculated based on the XRD results was 30–32 nm. The smaller particle size in comparison with the crystallite size shows that the TiO_2_ NPs sample had a developed specific surface area thanks to the presence of pores. 

It must be noted that the obtained size results, both for the calculations of the particle size and of the crystallite size, were calculated with the assumption that all particles were identical in terms of shape and size, which was not the case here as shown by the SEM and TEM results. 

The measurement of the average particle size of the TiO_2_ NPs suspension in the MMA monomer indicated the presence of TiO_2_ NPs agglomerates/aggregates with the average size of 3589 nm after 60 s of manual mixing, while after the application of the ultrasonic homogenisation process the average agglomerate/aggregate size increased to 4080 nm (Table 4). When comparing the polydispersity index, it is evident that the ultrasonic homogenisation process causes an increase in the suspension heterogeneity.

### 3.2. EDS Analysis 

**Figure 4:**
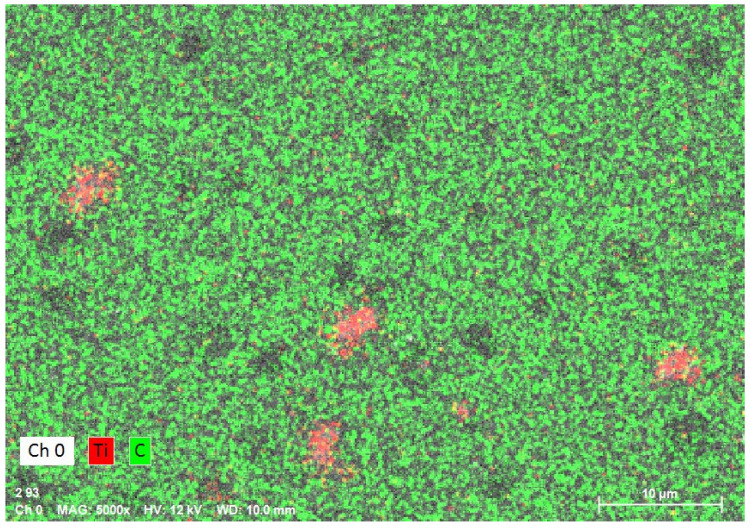
Element map of the nanocomposite for Ti (TiO_2_ NPs Cp = 1 wt%) using energy-dispersive X-ray spectroscopy (EDS) on SEM (nanocomposite manufactured without the sonication process).

**Figure 5:**
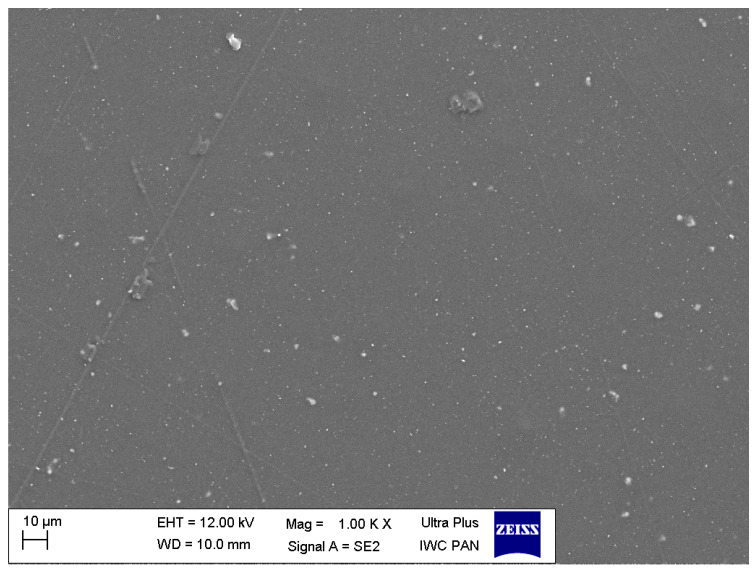
Representative SEM image of the nanocomposite (TiO_2_ NPs Cp = 1 wt%).

**Figure 6:**
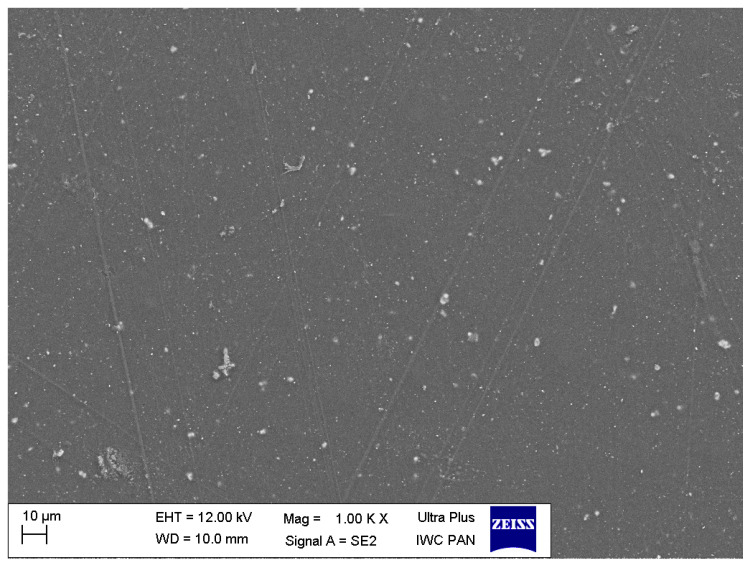
Representative SEM image of the nanocomposite (TiO_2_ NPs Cp = 2 wt%).

**Figure 7:**
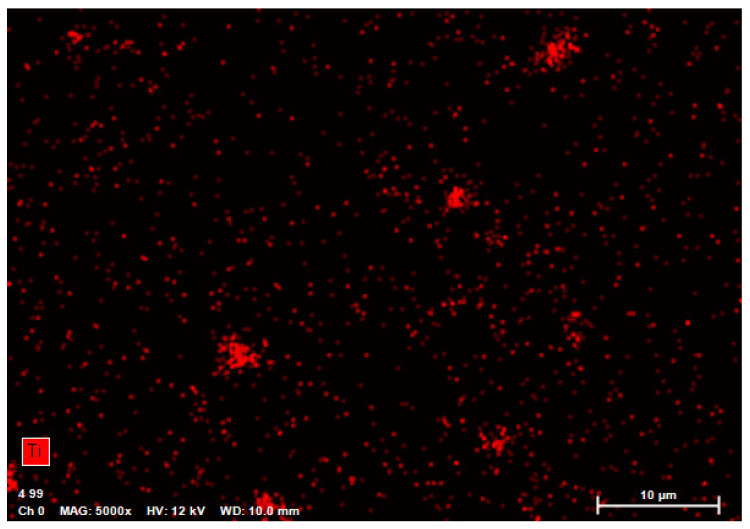
Element map of the nanocomposite for Ti (TiO_2_ NPs Cp = 2 wt%) using EDS on SEM.

**Figure 8:**
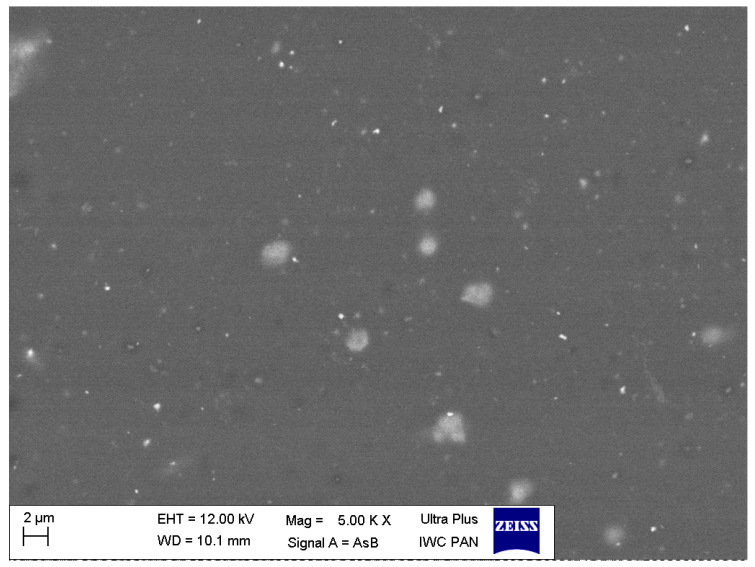
Representative SEM image of the nanocomposite (TiO_2_ NPs Cp = 2 wt%).

**Figure 9:**
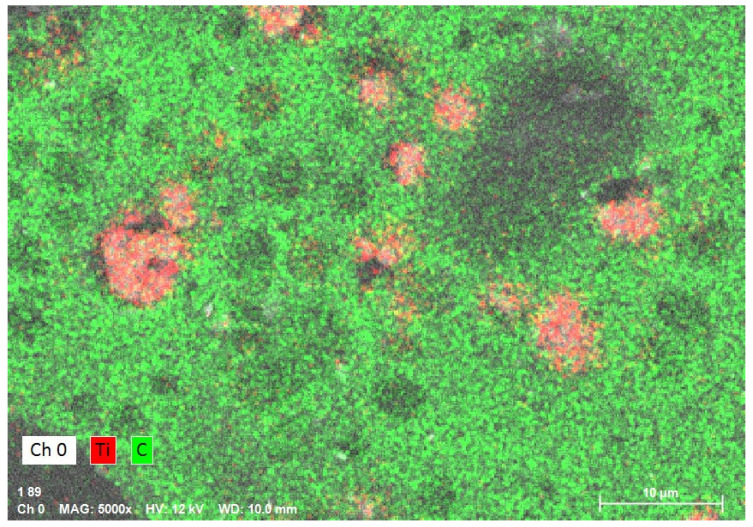
Element map of the nanocomposite for Ti (TiO_2_ NPs Cp = 1 wt%) using EDS on SEM (nanocomposite manufactured with the sonication process).

The sample mapping with the use of the EDS analysis revealed the presence of titanium particles in the composites, which formed, among others, conglomerates with the size reaching several micrometres (Figure 4). The increase in the concentration of the nanoparticles in the composite entails an increase in the cross-linking and density of distribution of titanium particles (Figure 5 and Figure 6). In addition, with the use of a higher concentration of the nanoparticles, a trend of an increased number of microcracks in the composite structure was observed. Despite the fact that titanium dioxide in the composite structure forms mainly conglomerates, it is at the same time present over the whole sample volume, where structures smaller than 100 nm can be observed, which constitutes a ground for calling the formed biomaterial a nanocomposite (Figure 7 and Figure 8). Simultaneously, when comparing representative SEM images of the samples created with the use of ultrasounds, no significant decrease in the quantity or size of TiO_2_ NPs conglomerates was observed (Figure 4 and Figure 9). 

### 3.3. Initial Polymerisation Time of Modified PMMA

**Figure 10:**
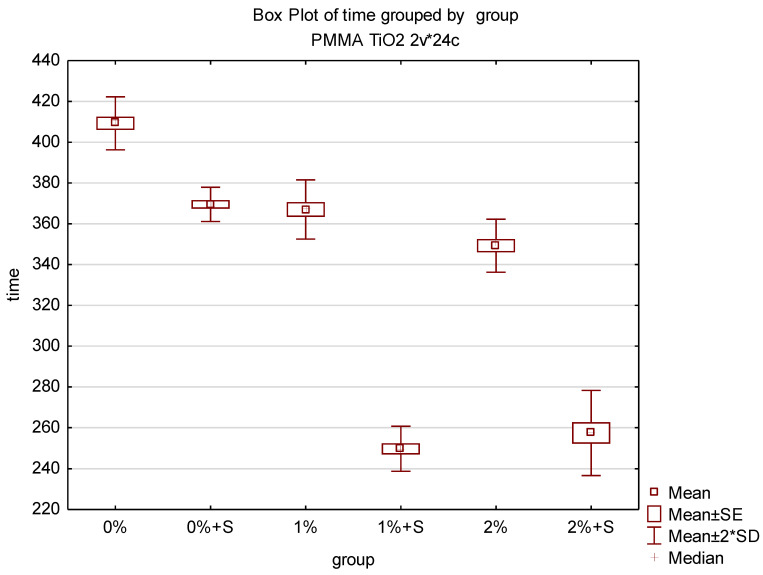
Box plot of the polymerisation time of nanocomposites.

Time measured from the moment of powder addition to the liquid until the moment of finishing the phase when the material adhered to the tool or vessel differed considerably among groups and the lowest value of 245 s was achieved for test groups 3 and 5, while the highest value of 415 s was achieved for the control group (group 0) The results of the time measurement are presented in Table 5. Despite the lack of modification of the acrylic material in group 0 and 1, the introduction of the sonication to the procedure of bonding resulted in a reduction of the average material polymerisation time and the difference between the groups was statistically significant. The incorporation of TiO_2_ NPs into PMMA reduced the average sample polymerisation time compared to the unmodified acrylic material and the differences between the control group and group 2 and 4 were statistically significant. The application of sonication procedure in the test groups containing TiO_2_ NPs reduced the average polymerisation time even more and the differences between the control group and group 3 and 5 were statistically significant. The results of the statistical analysis are presented in Table 6 and illustrated in Figure 10. 

### 3.4. Comparison of Colour of Modified and Unmodified PMMA

**Figure 11:**
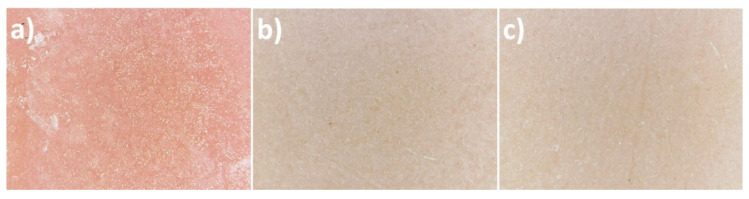
Photographs with the 60× magnification of the control group (**a**) and the test groups: 1% nanocomposite (**b**), 2% nanocomposite (**c**)**.**

The used samples of the prepared materials differed in colour and the difference was particularly evident between the control group sample and the samples of the material with incorporated TiO_2_ NPs. The photographs of the control group and the 1% and 2% nanocomposite with the 60× magnification are presented in Figure 11. The results of the colour measurements are provided in Table 7. The calculations of the colour distance in the CIE L*a*b* model between individual measurements indicate the greatest difference between the first measurement of the control group and the first measurement of group 2 (ΔE = 25.238). The lowest values of the colour change were recorded between individual measurements of the control group and between individual measurements of test groups 2 and 4, with the lowest value of ΔE = 0.352 between the first and third measurement of group 2. ΔE results at approx. 2 and below are designated as perceptively indistinguishable. The values of colour measurements in the RGB (R—Red, G—Green, B—Blue) colour space model indicate an increased red constituent and lower green and blue constituents in the sample obtained without modification with TiO_2_ NPs compared to the modified groups. Samples from group 2 and 4 in all measurements displayed a lower spread between the constituents of the RGB model, which translates into a more toned down output colour close to grey (milky), but still with the dominant red colour (highest values in the R constituent). The ΔE distance results in the three-dimensional CIE L*a*b* space between individual colour measurements are presented in Table 8 together with the colour designation of the results close to 2 (light green) and the results deviating from the perceptively indistinguishable difference (dark green).

## 4. Discussion

According to literature data [22] and the producer’s material safety data sheet [23], the sample of TiO_2_ NPs with the catalogue number 637254-50g that we purchased should only contain TiO_2_ in the form of anatase. However, the XRD analysis proved that the TiO_2_ lot (637254-50g) that we purchased contained an impurity in the form of rutile. Noman et al. [24] and Nyamukamba et al. [25] described in detail the crystalline structure of anatase and rutile and the differences in their properties in their overview papers. The results of a morphology test of a sample of TiO_2_ with the catalogue number 637254 carried out by Vicari et al. [26] confirmed our presumption that anatase was spherical/oval particles. Despite the presence of impurity being rutile in the TiO_2_ NPs sample, the specific surface area result obtained by us (81.6 m^2^/g) was very similar to the test results obtained by Vicari et al. [26] (83.5 m^2^/g), who used an analogous sample of TiO_2_ with the catalogue number 637254 in their tests. According to the producer’s data, the specific surface area of the TiO_2_ NPs sample that we purchased should range from 45 m^2^/g to 55 m^2^/g [23], which is reported also, e.g., by Fiorenza et al. [27] in their paper. The paper by Zaki et al. [28] reveals, in turn, that the TiO_2_ sample with the catalogue number 637254 that they used was characterised by the specific surface area of as much as 200–220 m^2^/g. The lack of repeatability of parameters of nanomaterials is a known issue [29], which may result from the unrepeatability of synthesis methods, actual changes of properties of nanomaterials (among others, product ageing), incorrect product label or unrepeatability of test procedures. Therefore, it is very important to perform tests of nanomaterials according to standardised test procedures and, if possible, at Accredited Laboratories.

Allowing dental practices and prosthetic laboratories to use the new material to a broad extent requires learning the physicochemical characteristics and the production methods in addition to their biological properties. Chemically polymerised materials are often used for relining dental prostheses in the case of loss of retention and stabilisation of the denture. The prosthesis relining procedure is also one of the methods of treating denture stomatitis by improving the quality of denture base adherence to the mucous membrane and eliminating the action of the mechanical injury [11]. Such a procedure decreases the number of microorganisms present in the outermost material layer and also permits the addition of an antifungal or antibacterial substance to the relining material. The introduction of TiO_2_ to materials applied in prosthodontics seems reasonable due to the microbiological activity of titanium nanoparticles [30,31]. The photocatalytic properties of titanium oxide are well known and broadly described; in presence of water and a close range of UV light (λ ≤ 387 nm) it generates reactive forms of oxygen preventing the development of pathogens and the formation of biofilm [31]. Meng et al. [32] highlight at the same time that the antibacterial action may be intensified by the modification of TiO_2_ itself with such metals as Fe, thus increasing the activating spectrum to the visible light range. The effectiveness of the antimicrobial action is correlated by the authors with the weight content of titanium oxide in relation to PMMA [32]. Cheng [33] provides the weight content of 5% as optimum in terms of microbiology. Reportedly, even the 0.4% content displays an action that inhibits colonisation with biological, both bacterial and fungal, material [34]. On the other hand, in other research, the 0.8 wt% content of nano TiO_2_ did not cause inhibition of biofilm formation on the PMMA surface [15]. Therefore, this publication assumes that the TiO_2_ NPs weight content of 1% and 2% are values with a documented antimicrobial action and at the same time with the minimised possibility of a negative effect on other properties of the material [6,35].

There are many studies describing methods of preparation of the PMMA-TiO_2_ hybrid material [6]. The two primary methods of preparation of these materials are the incorporation of titanium particles into the whole poly(methyl methacrylate) volume and coverage of the surface of ready PMMA with a TiO_2_ layer [6,34,35,36]. In this paper, the authors propose to use a hybrid of a self-curing polymer and TiO_2_ NPs as a material among others for prosthesis relining and thereby to change the properties of the layer being in direct contact with the oral mucosa. The described procedure uses a relatively simple scheme of material preparation with the use of an ultrasonic cleaner to enable its possible repetition in dental practices. The application of ultrasounds was hypothetically supposed to increase the dispersion of nanoparticles in PMMA. The DLS test of TiO_2_ nanoparticles in a liquid acrylic resin monomer indicated an increased size of conglomerates and when comparing the polydispersity index it was found that the ultrasonic homogenisation process caused an increase in the suspension heterogeneity. The analysis of SEM images of the obtained biomaterial showed a tendency of formation of TiO_2_ NPs conglomerates of various sizes, and their distribution over the compound proved independent of the adopted sample preparation procedure (with the use of an ultrasonic cleaner or only with manual mixing). Dispersion in PMMA resins may be impeded due to Van der Waals forces occurring between TiO_2_ NPs [37]. However, the formation of such kind of conglomerates is to be avoided, in particular in view of the mechanical, e.g., tensile, strength. The application of ultrasounds was not relevant when comparing representative SEM images. With the use of a higher concentration of the nanoparticles, a trend of an increased number of microcracks in the composite structure was observed. These observations are consistent with the data reported by Moslehifard et al. [37] and also by Alrahlah et al. [38], who state in the conclusions of their papers that the 1% nanocomposite indicated a lower number of microcracks and a reduced flexural strength compared to the 2% nanocomposite. Despite the positive impact of TiO_2_ content in the PMMA structure on the results of stiffness or tensile tests (35% better result for 1 wt% TiO_2_ NPs than for pure PMMA), it was observed in the tests that bigger clusters of TiO_2_ NPs in the PMMA matrix act like an impurity of the polymer matrix and are centres of reduced stress strength [39,40]. Thus, it is reasonable to establish a protocol that improves the distribution of non-clustered TiO_2_ NPs in the PMMA mass both in biological and mechanical terms [39].

The authors did not encounter such research in the available literature that concerned polymerisation time depending on the application of TiO_2_ nanoparticles or ultrasounds. This is of enormous clinical importance because a hypothetical considerable reduction in the time of work with a given biomaterial may significantly impede or prevent the creation of a dental prosthesis. Nazirkar et al. [41] reported a reduction of flexural strength values of TiO_2_-PMMA nanocomposite compared to the conventional unmodified resin. The authors stated that the introduction of reinforced TiO_2_ NPs could interfere with the polymerisation process but they failed to explain the possible mechanism of this action. Tandra et al. [42] observed that the formation of greater quantities of TiO_2_ NPs agglomerates interfered with the polymerisation reaction. TiO_2_ NPs additives at high concentrations also increased the amount of residual monomer by acting as a plasticiser [43]. The plasticising effect leads to a reduction of the monomer conversion rate and high amounts of residual monomer, which could change the polymerisation time of the new biomaterial [44]. It is proved based on this publication that the time of initial polymerisation of poly(methyl methacrylate) decreases in line with the increase in the weight concentration of TiO_2_ NPs in mass and it may be reduced even more by accelerating factors such as temperature increase, energy supply, or chemical catalysts. Chatterjee provides [36] that due to the presence of a hydroxyl group (–OH) on the TiO_2_ NPs surface and a carbonyl (C=O), hydroxyl (–OH), carboxyl (–COOH) and ester (–COOR) group in the polymer matrix, there is a possibility of a chemical connection between them in two manners: through hydrogen bonds and coordinate bonds with the Ti^4+^ cation. The effect of such connections is increased cross-linking of the PMMA matrix in the vicinity of the TiO_2_ NP. It is not excluded that it is the cross-linking capacity of the thermosetting polymer that decreases the initial polymerisation time in this research. Among all the conducted trials, the shortest polymerisation time was 245 s, which is certainly sufficient for the performance of all technical procedures when relining, repairing or even making new dental prostheses.

Titanium dioxide is widely applied as a pigment in numerous industry sectors and in medicine. This compound is used as a pigment also by producers of dentistry polymer materials used for making prostheses [30]. The obtained data reveal that the addition of both 1 wt% and 2 wt% causes a considerable change in the PMMA colour: its whitening. Our research reveals that a colour change between pure PMMA and PMMA-TiO_2_ NPs is considerably more conspicuous than between 1 wt% PMMA-TiO_2_ NPs and 2 wt% PMMA-TiO_2_ NPs. The decreased transparency of the formed material is caused by a high refractive index, by which TiO_2_ NPs are characterised. The shape and size of TiO_2_ NPs also permit filling microspaces in the PMMA polymer matrix, which on the one hand positively affects material polishability and on the other hand should potentially decrease material absorbability, and hence, its swelling [40,45,46]. Permeation of water particles into the polymer chain grid causes the occurrence of internal tensions and propagation of microcracks, as a result of which polymer materials may become discoloured. Gad and Abualsaud [6] provide in their literature review that an improvement of mechanical properties without a negative impact on aesthetics would be a perfect feature of the reinforced material. This is particularly important when the whole denture base is made of the PMMA-TiO_2_ NPs material. From the point of view of the presented study, where the obtained material should be used mainly as a relining material, hence present on the impression surface of the denture base, the microbiological and mechanical issues seem more important than obtaining appropriate transparency. In the subjective opinion of the authors, even a 1% nanocomposite as a material that restores the pink aesthetics in dental prostheses may encounter significant problems of aesthetic nature and will not be accepted by those patients for whom biomimetics and natural appearance are a priority. 

## 5. Conclusions

1. The presence of TiO_2_ nanoparticles sized below 100 nm was indicated in the structure of the newly created biomaterial, which constitutes a ground for calling it a nanocomposite.
2. There is no justification for the use of ultrasonic cleaners in the procedure of TiO_2_ nanoparticles incorporation into PMMA.
3. The reduced polymerisation time of the new biomaterial fully enables the performance of standard procedures related to the creation of denture bases.
4. Due to the considerable change in the colour, the clinical application is limited to the performance of repairs or relining of the prosthesis, where the new material is located in an area with no aesthetic requirements (impression surface or not exposed polished surface of the denture base).

## Figures and Tables

**Table 1:** Composition of particular nanocomposites.

Composition	PMMA (Control Group)	1% Nanocomposite	2% Nanocomposite
TiO_2_ NPs powder	0 g	0.32 g	0.64 g
PMMA powder polymer	22 g	22 g	22 g
Liquid monomer of PMMA	10 g	10 g	10 g

**Table 2:** Characteristics of groups during the initial polymerisation time test.

Group	Group Designation	Number of Trials	TiO_2_ NPs Content	Manual Mixing	Use of Ultrasounds
0 (control group)	0%	4	0%	Yes	No
1	0%S	4	0%	Yes	Yes
2	1%	4	1%	Yes	No
3	1%S	4	1%	Yes	Yes
4	2%	4	2%	Yes	No
5	2%S	4	2%	Yes	Yes

**Table 3:** Characteristics of the TiO_2_ NPs.

Sample	Specific Surface Area, a_s_ (m^2^/g)	Skeleton Density, ρ_s_ ± σ (g/cm^3^)	Average Particle Size from SSA BET, d ± σ (nm)	Average Crystallite Size from Nanopowder XRD Processor Demo, d ± σ (nm)	Average Crystallite Size, Scherrer’s Formula, d ± σ (nm)
TiO_2_ NPs	81.6	3.68 ± 0.01	20 ± 1	30 ± 8	32 ± 20

d—diameter.

**Table 4:** Average diameter of TiO_2_ NPs in a methyl methacrylate suspension, Cp = 0.01 wt%.

Sample TiO_2_ NPs/MMA	Size by DLS, Z-Average, d ± σ (nm)	Polydispersity Index, Pdl
Mixing for 1 min	3589 ± 914	0.349 ± 0.052
Mixing for 1 min and ultrasonic mixing for 240 s and ultrasonic mixing for 60 s	4080 ± 567	0.562 ± 0.227

**Table 5:** Characteristics of groups during the initial polymerisation time test.

Group	Trial	Initial Polymerisation Time (s)	Mean	SD
0 (0%)	1	400	409.25	6.5
	2	410		
	3	415 (highest)		
	4	412		
1 (0% + S)	1	375	369.5	4.20
	2	370		
	3	410		
	4	390		
2 (1%)	1	375	367.0	7.26
	2	370		
	3	358		
	4	365		
3 (1%S)	1	245 (lowest)	249.75	5.50
	2	255		
	3	245 (lowest)		
	4	254		
4 (2%)	1	340	349.25	6.50
	2	350		
	3	355		
	4	352		
5 (2%S)	1	260	257.5	10.41
	2	255		
	3	270		
	4	245 (lowest)		

**Table 6:**
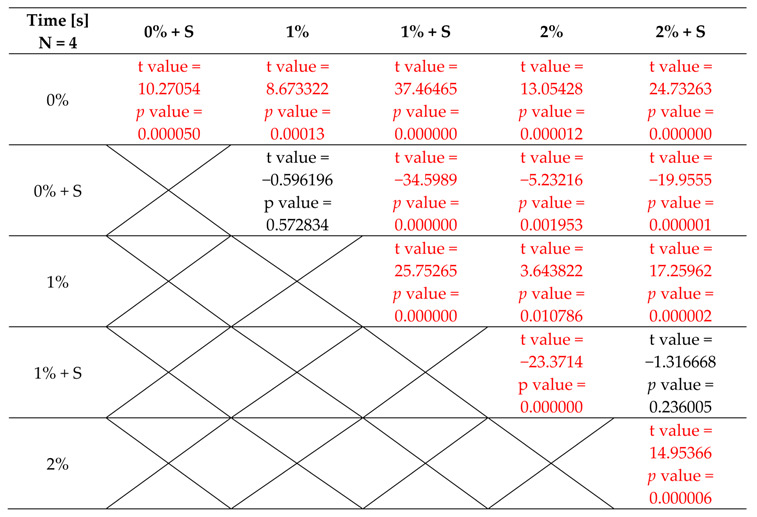
Results of the polymerisation time of nanocomposites.

**Table 7:** Nanocomposite colour analysis.

Group	Measurement	CIE L*a*b* Colour Model	RGB Colour Model
0 (0% TiO_2_ NPs)	1	L 44.13 a 9.92 b 3.62	123.98.99
	2	L 44.64 a 9.98 b 3.69	124.100.100
	3	L 44.36 a 10.02 b 3.30	124.99.100
2 (1% TiO_2_ NPs)	1	L 66.98 a 10.90 b 14.29	192.155.138
	2	L 65.14 a 10.69 b 12.46	185.151.136
	3	L 66.67 a 10.99 b 14.15	191.155.137
4 (2% TiO_2_ NPs)	1	L 67.68 a 10.75 b 12.64	193.158.143
	2	L 65.60 a 10.59 b 13.25	187.152.136
	3	L 66.54 a 10.49 b 12.42	189.155.140

**Table 8:**
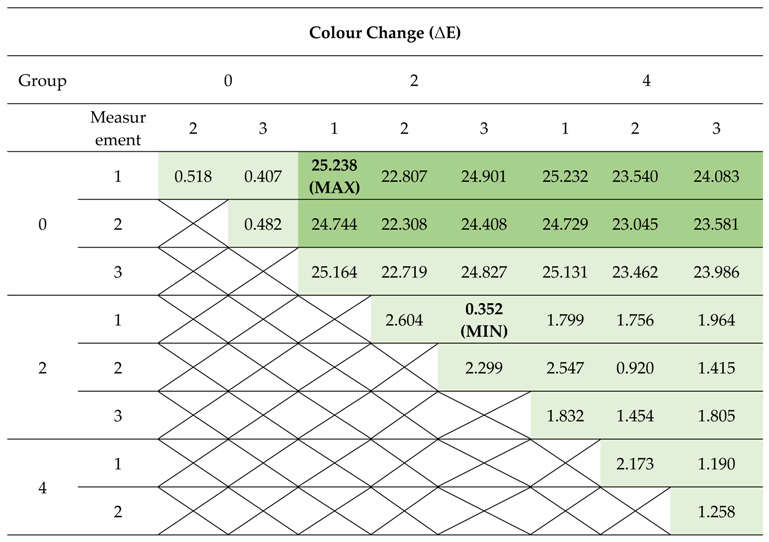
Nanocomposite colour change analysis.
